# A novel cardio-oncology service line model in optimizing care access, quality and equity for large, multi-hospital health systems

**DOI:** 10.1186/s40959-023-00167-0

**Published:** 2023-03-27

**Authors:** Yan Liu

**Affiliations:** 1 Cardio-Oncology Service Line, Ascension Texas, Austin, USA; 2 Cardio-Oncology, Institute for Cardiovascular Health, UT Health Austin/Ascension, Austin, USA; 3 Dell Medical School, University of Texas at Austin, 1004 W 32nd St #300, Austin, TX 78705 USA

## Abstract

**Background:**

Despite the rapid growth of cardio-oncology as a subspecialty, cancer patients are still underserved from a cardiovascular perspective. A new care model is needed to integrate comprehensive cardio-oncology care with community-based facilities to improve care access, quality, and equity. Here, we present a cardio-oncology service line model for large, multi-hospital health systems to address this need.

**Methods:**

An academic cardio-oncology program was first established using a multidisciplinary approach. Five infrastructure elements for a service line model were created, including strategic accountability, standardized care, dedicated resources, patient experience/education, and branding/identity. We then utilized these elements across our healthcare system to establish a quality-controlled and centrally governed cardio-oncology service line structure. Protocols were created to standardize care and ensure consistency and quality, including referral workflow, imaging, cardiotoxicity surveillance, and clinical management. An IRB-approved cardio-oncology registry was established for outcome tracking.

**Results:**

The standardized cardio-oncology services were expanded to eight hospitals and ten outpatient care centers, including rural outreach offices, resulting in increased patient access and improved clinical quality measures. The service area expanded 17-fold, and an estimated rural population of 204,133 gained access to care. Cardio-oncology office visits increased by approximately 600% three years after implementation of the service line model.

**Conclusions:**

A cardio-oncology service line with standardized care is a feasible and effective care model to improve cardio-oncology care quality, patient access, and health equity in large, multi-hospital health systems. It can be used in conjunction with academic cardio-oncology programs to improve the overall cardio-oncology healthcare efficacy in the US.

## Background

Cardiovascular disease (CVD) and cancer are the top two causes of mortality in the US and worldwide [1, 2] despite advances in prevention and treatment. There are currently an estimated 16.9 million cancer survivors in the US, and this number is expected to grow to over 22.1 million by 2030 [3]. Cancer and cardiovascular comorbidities and toxicities are leading causes of morbidity and mortality in cancer patients and survivors [4–6]. While cardio-oncology services are becoming more established in academic centers and local communities, cardio-oncology patients remain underserved from a cardiovascular care standpoint, despite emergent data demonstrating that cardiovascular treatment can improve both cardiac-specific and cancer-specific outcomes [7–9]. This care access and equity gap is particularly critical in rural populations, where access to essential healthcare services is limited and cancer mortality rates are higher than in urban areas [10, 11]. It is essential to build a care model that integrates comprehensive and standardized cardio-oncology care with community-based hospitals and practices to optimize patient access and health equity. We present a novel cardio-oncology service line model for large, multi-hospital health systems in collaboration with academic cancer centers and local community oncology practices to address this important need.

## Methods and results

**Fig. 1:**
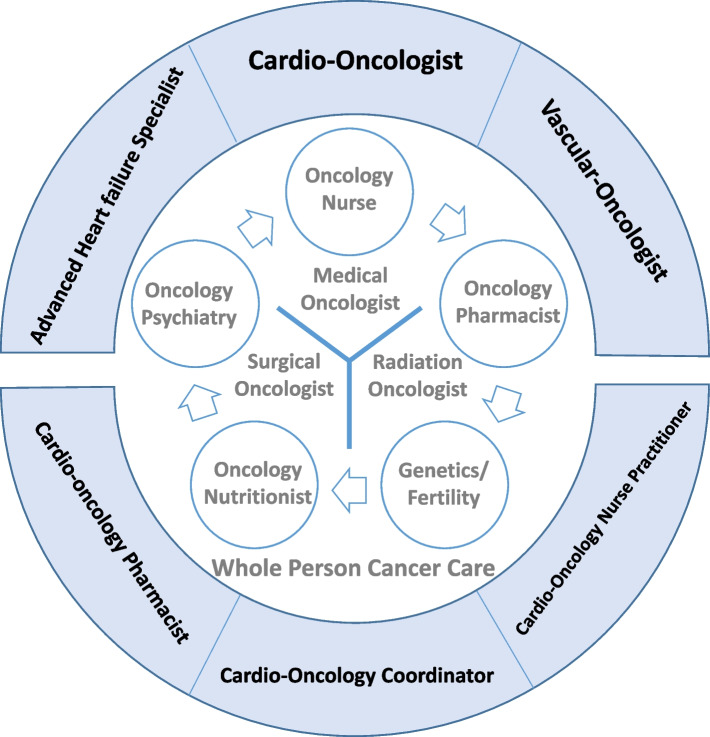
An academic cardio-oncology program that was established in partnership with a cancer center, with a focus on multidisciplinary collaboration, whole person care, and optimized patient outcomes. The program provides comprehensive cancer care, addressing all medical and supportive care needs, through a team of experts, including cardio-oncologists, vascular oncologists, specialists in advanced heart failure and infiltrative heart disease, a cardio-oncology pharmacist, a cardio-oncology administrative coordinator, and nurse practitioners. The cardio-oncology team works in close collaboration with the oncology team, with direct and efficient communication established through a cardio-oncology hotline and an encrypted designated email address. This enables real-time updates on the status and management of cardio-oncology patients. Overall, the program prioritizes the needs of the whole person, recognizing the complex interplay between cancer and cardiovascular health, and seeks to optimize patient outcomes through a collaborative, patient-centered approach

The academic cardio-oncology program was initially established in partnership with a cancer center, with the core principles of multidisciplinary collaboration, a focus on whole-person care, and an objective of optimizing overall patient outcomes (Fig. 1). The comprehensive cardio-oncology team comprises a range of healthcare professionals, including cardio-oncologists, vascular oncologists, specialists in advanced heart failure and infiltrative heart disease, cardio-oncology pharmacists, cardio-oncology administrative coordinators, and nurse practitioners (Fig. 1). The team ensures whole-person cancer care, and facilitates direct and efficient communication with the oncology team through a cardio-oncology hotline and an encrypted email address, enabling real-time updates on the status and management of cardio-oncology patients.

**Fig. 2:**
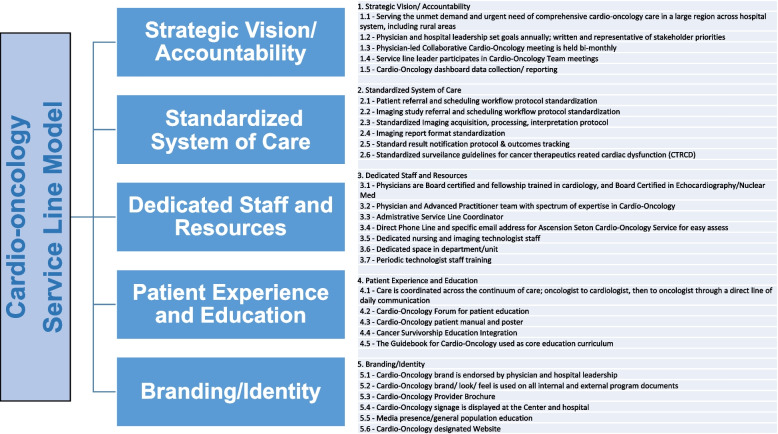
The five key infrastructure elements of the cardio-oncology service line model: strategic vision and accountability, standardized system of care, dedicated staff and resources, patient experience and education, and branding and identity. These elements were developed and implemented across multiple hospitals and offices within our healthcare system to establish a system-wide cardio-oncology service line. The goal of this service line model is to create a quality-controlled, evidence-based, and centrally governed structure that provides optimal care for cardio-oncology patients

To develop the cardio-oncology service line, we defined and implemented five key elements of infrastructure, including strategic vision and accountability, standardized system of care, dedicated staff and resources, patient experience and education, and branding and identity (Fig. 2). These elements were implemented across our healthcare system to expand the cardio-oncology service, with a focus on forming a quality-controlled, evidence-based, and centrally governed service line structure.

Our strategic vision was established to address the unmet demand and urgent need for comprehensive cardio-oncology care both within and beyond our healthcare system. Strategic accountability was enforced through goal-directed efforts from both health system and cardio-oncology leadership. Bimonthly service line meetings were held system-wide, led by the cardio-oncology director and attended by health system and physician group leadership, to address needs and challenges. To support data collection and reporting, a dashboard based on an IRB-backed cardio-oncology registry was implemented (Fig. 2).

During the three-year service line development, dedicated staff and resources were gradually established. This included multiple cardio-oncology physicians, such as cardio-oncologist/imaging specialists, a vascular oncologist with a focus on vascular and coronary intervention, and an advanced heart failure/infiltrative heart disease specialist. Additionally, two cardio-oncology nurse practitioners, one cardio-oncology pharmacist, and an administrative nursing coordinator were brought on board. It's worth noting that the service line was established during the pandemic, which posed unique challenges in recruiting and hiring staff.

To provide comprehensive cardio-oncology care, a dedicated clinical and administrative space was created with cardio-oncology signage prominently displayed. This helped patients feel more at ease and supported as they navigated the challenges of their cancer and cardiovascular conditions. The service line was also equipped with designated echocardiogram technologists who were experienced in 3D and strain echocardiography, and who received periodic training across the health system based on a newly created cardio-oncology echocardiogram protocol (Fig. 2).

**Fig. 3:**
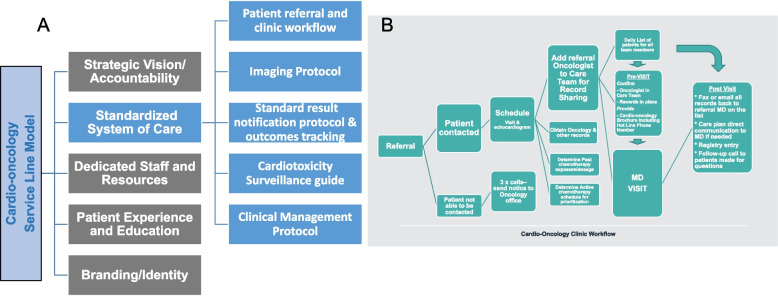
The standardized system of care as the foundation of the service line model. **A** five essential protocols for care standardization are highlighted, demonstrating the emphasis on evidence-based care and the need for consistency in care across all healthcare facilities. These protocols are crucial to ensuring that all patients receive the same high-quality care, regardless of where they receive treatment within the healthcare system. **B** Cardio-oncology patient referral and clinical workflow is illustrated. This workflow was utilized in all branch offices to ensure timely scheduling of all referred patients. The workflow emphasizes a detail-oriented, step-by-step, close-end communication process to ensure that all records and recommendations are shared with the referring oncologists and providers once they become available. This ensures that all healthcare providers involved in the patient's care are informed and able to make informed decisions based on the patient's needs

**Fig. 4:**
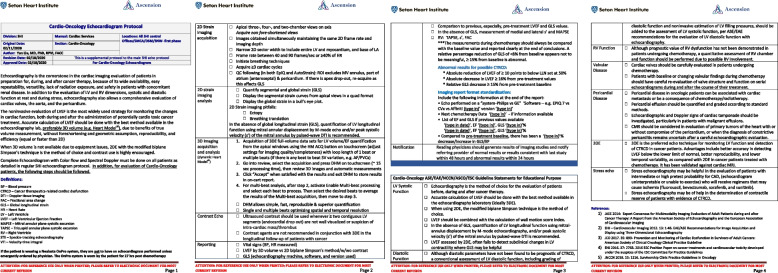
Cardio-oncology echocardiogram protocol. With 3D left ventricular ejection fraction and global longitudinal strain as the major highlights, the cardio-oncology echocardiogram protocol was created to standardize image acquisition, reporting and notification process. The cardio-oncology echocardiogram protocol was also equipped with an educational component. The protocol was implemented in all eleven echocardiogram labs with periodic and repeated training of echocardiogram technologists across health system

The foundational element of developing a service line model was the establishment and implementation of a standardized system of care across our multi-hospital organization. Over a three-year period, we developed five essential protocols for care standardization (Fig. 3A). We successfully established and refined our cardio-oncology patient referral and clinical workflow (Fig. 3B) to be used in all ten branch offices, prioritizing patients undergoing active chemotherapy for timely scheduling. Our workflow emphasized a detail-oriented, step-by-step, close-end communication process to ensure all records and recommendations were communicated back to referring oncologists and providers once available. The second component of our standardized system of care is a system-wide imaging protocol (Fig. 4) for cardio-oncology echocardiogram, with 3D left ventricular ejection fraction and global longitudinal strain as the major highlights. This protocol standardizes image acquisition, reporting, and notification processes. Furthermore, the cardio-oncology echocardiogram protocol includes an educational component and is used in periodic and repeated training of echocardiogram technologists across our health system. The third component of our standardized system of care is a standardized scheduling and result notification protocol and outcome tracking across our institution (Table 1). This protocol serves as a guideline for all cardio-oncology related result notifications, including consultations, imaging, and procedures. It ensures consistency and reliability in our cardio-oncology care system.

**Table 1:** The standardized cardio-oncology patient scheduling and test result notification protocol. Established across our institution, this protocol serves as a comprehensive guideline and quality control mechanism for all cardio-oncology related scheduling and result notification processes, including consultation, imaging, and procedures. By providing consistency and reliability for patient care, this protocol ensures the highest quality of care for cardio-oncology patients

2. Standardized Scheduling and Result Notification Protocol
2.1—Patient referral and Imaging study workflow protocol standardization
2.1.1—Dedicate appointment slots in NIV imaging department to accommodate timely Strain Echo testing
2.1.2—Provide oncology offices a Cardio-Oncology order AID for direct scheduling capabilities for Cardio-oncology echos/peripheral studies with instruction to call central scheduling for all appointment requests (including urgent requests)
2.1.3—Cardio-Oncology patients should be scheduled for timely NIV imaging studies (in < 5 business days)
2.1.4—Cardio-Oncology patients should be scheduled for timely clinical visits (in < 10 business days)
2.1.5—New Cardio-Oncology referrals need echo prior to or on the day of visit (if no echo within last 4 weeks)
2.1.4—Participating Cardiologists should dedicate outpatient clinic appointment slots for cardio-oncology patients
2.1.5—Information to be obtained from Ordering oncology offices
* Type of Chemo, frequency, and dose
* Treatment start date
* Date of next scheduled appointment
* Name of ordering physician, contact information, & Fax #
2.2—Standardized Imaging acquisition protocol on approved software platforms
2.2.1—Sonographers are to reference our NEW Cardio-Oncology Echocardiogram Protocol
2.2.2—Vendors software and tech training for 3D/strain echo
2.3—Imaging report format standardization
2.3.1—Include the following information in the report, per ASE guidelines:
• Echo performed on a ‘(type in) system’ version ‘(type in)’
• ‘(type in date)’, EF ‘(type in)’, GLS ‘(type in)’%
• On this study, there has been a ‘(type in)’% change in GLS compared to pretreatment baseline
2.4—Standard result notification protocol & outcomes tracking
2.4.1—Reading physicians should generate results of imaging studies and notify referring provider of normal results or results consistent with last study within 48 h via fax/Athena
2.4.2—Reading physicians should generate results of imaging studies and notify referring provider directly or on-call MD to explain abnormal results within 24 h (abnormal results include but not limited to CTRCDs
2.4.3. Evidence of CTRCDs, Per ASE guidelines, abnormal results for chemotoxicity:
* Absolute Reduction of LVEF of > 10 points to below LLN set at 50%
* Absolute decrease in LVEF > 16% from pre-treatment values
* Relative GLS decrease > 15% from pre-treatment baseline

The standardized system of care includes a fourth component, which consists of a therapy-specific cardiotoxicity risk and surveillance guide (Tables 2 and 3), based on guidelines and literature. In the fifth component, we have developed a clinical management protocol for cardiotoxicity based on the most recent ESC cardio-oncology practice guidelines [12] for both outpatient and inpatient settings. These components are regularly updated every two months during cardio-oncology service line meetings to ensure that the latest evidence is being utilized. These service line meetings not only serve as an opportunity for updating and improving the care system but also as an educational platform for all members of the cardio-oncology team across the health system.

**Table 2:** The cardio-oncology surveillance guide, which outlines the cardiac surveillance and prevention protocols for both inpatient and outpatient settings. Shown here is a part of a comprehensive cardio-oncology protocol that was developed based on the most updated practice guidelines. Please note that the complete version of the protocol is not shown here

I. Cardiotoxicity Surveillance and Prevention
High Risk for Cardiotoxicity
* Age > 60
* (> = 2) PMH: HTN, HLD, DM, CKD2, FH of cardiomyopathy/premature CAD
* (> = 2) PSH: Smoking, alcoholism, obesity, sedentary lifestyle
* Cardiac: pre-existing CAD, arrhythmia or structural heart disease
* Prior exposure: > = 250 mg/m2 cumulative doxorubicin dose or equivalent
* Prior exposure: prior chest/mediastinum RT
* Prior exposure: Sequential anthracyclines + trastuzumab
* Timing of exposure: childhood cancer survivors with exposure
Indications for Cardio-Oncology Consultation
Outpatient:
• Cancer therapy related cardiac dysfunction (CTRCD)
• Asymptomatic cardiac abnormality with cancer therapy
• Primary prevention in patients with CV toxicity risk factors
• Secondary prevention for chemo-induced cardiotoxicity
• Cancer patients or survivors with concurrent cardiovascular disease
• Pre-clinical trial cardiac optimization for cardiotoxic trial agents
• Cancer with cardiac involvement
• Infiltrative heart disease, e.g. Cardiac Amyloidosis
• Childhood cancer survivors
Inpatient:
o Any active cardiac issues in the setting of active/recent/planned cancer therapeutics or recent diagnosis of cancer
o No active cardiac issues but starting new chemotherapy with history of cardio-toxicity/ cardiomyopathy/cardiovascular disease/high risk as stated above
Surveillance and Monitoring Protocol after treatment in patients at risk for CTRCD (for anthracycline or trastuzumab)
- Pre-Treatment for all patients on anthracycline or trastuzumab
- During Anthracycline Treatment: at completion of therapy and 6 months later if < 240 mg/m2, or prior to treatment of each additional 50 mg/m2 if reach or exceeding 240 mg/m2. 6 months after Anthracycline Treatment: At the discretion of cardiologist & oncologist
- During Trastuzumab Treatment: Every 3—6 months (based on patient risk factors)
- After Trastuzumab Treatment: Every 6 months for two years post-treatment, and then at the discretion of cardiologist & oncologist after two years
To be continued.

**Table 3:** The cardiotoxicity risk reference for providers. This table serves as a valuable resource for healthcare providers involved in the care of cardio-oncology patients, helping them to identify and manage potential cardiotoxicity risks associated with cancer treatments

Cardiac dysfunction	Agents	Risk	Mechanism
Heart failure	Anthracyclines	Cumulative, high	Myocyte death
	Cyclophosphamide	Low	Myocarditis
	Cisplatin	Low	Unknown
	Trastuzumab	Moderate-high	Contractile protein dysfunction
	Lapatinib	Low	
	Bevacizumab	Low	Hypertension?
	Sunitinib	Low	Mitochondrial dysfunction
	Sorafenib	Low	
	Carfilzomib	Moderate	
	Imatinib	Low	Mitochondrial dysfunction
Arterial hypertension	All angiogenesis inhibitors/anti-VEGF/TKIs	Moderate, dose-dependent	Endothelial dysfunction
Myocardial ischemia	Pyrimidine analogues	Moderate	Direct vasospasm
Coronary heart disease	BCR-ABL TKIs(esp. Nilotinib, Ponatinib)	Moderate	Endothelial damage
Thromboembolism	Cisplatin, All angiogenesis inhibitors, anti-VEGFs, Bcr-ABL TKIs (Nolitinib, Ponatinib)	Moderate	Endothelial dysfunction
Arrhythmia/QT prolongation	Arsenic trioxide, TKIs, Proteasome inhibitors	Moderate	HERG K + blockage
Atrial Fibrillation	Ibrutinib (BTKi)	Moderate	PI3K-akt inhibition
Pulmonary arterial hypertension	Dasatinib	Moderate	HERG K + blockage
VT/VF/Complete HB Fulminant myocarditis	Immune checkpoint inhibitors	Rare with high mortality	Co-stimulatory pathway
Vascular Toxicity	BCR-ABL TKIs	Moderate	Endothelial dysfunction
Premature atherosclerosis	HSCT(Hematopoietic stem cell transplant)	Moderate	Endothelial damage and inflammatory response

Close collaborations have been established with internal and external referral providers, including oncology practices, cancer centers, primary care providers, and survivor clinic providers, to ensure that consistent care is provided in accordance with the standardized protocols. To monitor the outcomes, an IRB-approved cardio-oncology registry has been established. All protocols and guidelines have been made readily available to staff and providers across the healthcare system through a designated intranet link. These protocols and guidelines are updated periodically based on the latest evidence during the cardio-oncology service line meetings.

In building the cardio-oncology service line (Fig. 2) as a new specialty, we believe that the patient experience and education are critical elements. The coordination of patient care is ensured across the continuum of care, including communication between oncologists and cardiologists through a direct line of daily communication, utilizing a direct cardio-oncology phone line and a designated encrypted email address. Patients and their families are kept updated simultaneously to ensure informed shared decision making, and the same direct hotline is available for easy patient access. Our cardio-oncology service has also established a collaborative relationship with oncology infusion centers to address any urgent or unexpected needs.

We have developed and distributed a patient educational manual and hold periodic patient educational forums for active cancer patients and cancer survivors. The final element of the service line model is branding and identity (Fig. 2), which is essential for any new cardio-oncology program. We have established this through referral provider outreach and education, cardio-oncology provider educational brochures, CME-based educational events internally and externally, and a designated cardio-oncology website.

**Fig. 5:**
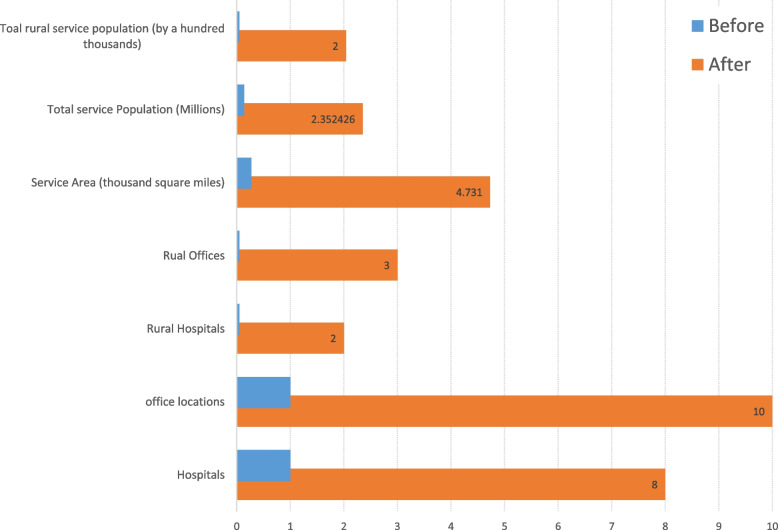
The positive impact of implementing a service line care model on patient access to cardio-oncology services. Following the establishment of the service line model, the standardized cardio-oncology services were expanded from one medical center to eight different hospitals across the hospital system, including two rural hospitals. Outpatient cardio-oncology care was expanded from one outpatient care center to ten different locations, including three rural outreach offices. Consequently, the cardio-oncology service area significantly increased from 274 square miles to an estimated 4731 square miles, providing access to a population of 2.4 million. Furthermore, the service line model facilitated cardio-oncology care for an estimated rural population of 204,133 who previously lacked access to these services

**Fig. 6:**
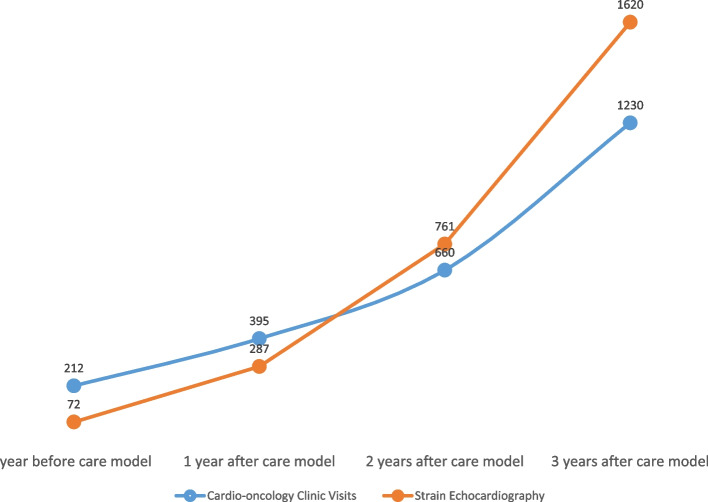
The significant impact of the service line care model on cardio-oncology clinic visits and strain echocardiography use. The implementation of the service line model resulted in an average 200% yearly increase in cardio-oncology patient visits, which eventually reached six times the initial volume within three years. Moreover, the appropriate utilization of strain echocardiography steadily improved year over year, leading to a remarkable over 20-fold increase in study utilization over the three-year period

After implementing the new cardio-oncology service line, the standardized cardio-oncology services expanded significantly, from being available at one medical center to eight hospitals across the hospital system, which included two rural hospitals (Fig. 5). Additionally, outpatient cardio-oncology care was expanded from one outpatient care center to 10 different locations, including three rural outreach offices (Fig. 5). Consequently, the cardio-oncology service area increased from 274 square miles to an estimated 4731 square miles (Fig. 5), providing access to a population of 2.4 million. The number of cardio-oncology patient visits increased by an average of 200% annually, which reached approximately six times higher three years after the service line model was established and implemented during the pandemic (Fig. 6). Moreover, strain echocardiography was more appropriately utilized, resulting in a 20-fold increase over a three-year period (Fig. 6).

**Fig. 7:**
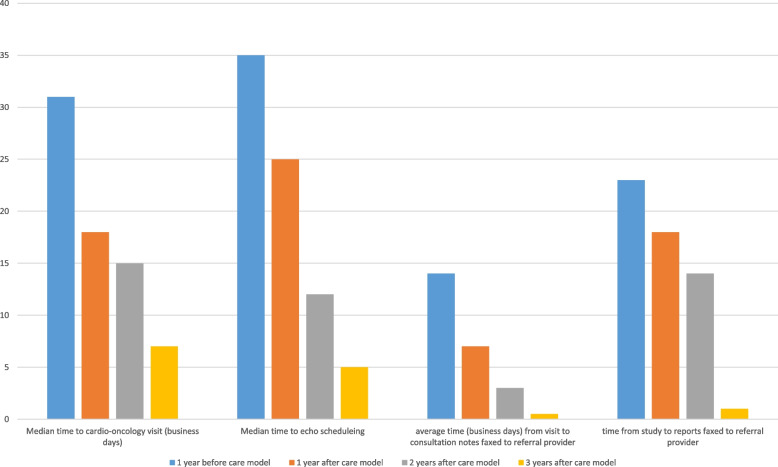
The substantial improvement in clinical flow quality measures following the establishment of the service line care model. These quality measures include the time from referral to office visit, time from referral to echocardiogram study, time from visit to consultation notes faxed to the referral provider, and time from study to reports faxed back to the referral provider. Notably, these measures have shown a significant year-over-year improvement when compared to the pre-standardization period

Furthermore, the establishment of the service line allowed cardio-oncology care to become newly accessible to an estimated rural population of 204,133 (Fig. 5). The quality measures, including time from referral to office visit, time from referral to echocardiogram study, time from visit to consultation notes faxed to the referral provider, and time from study to reports faxed back to the referral provider, have shown significant improvement when compared to before care standardization (Fig. 7). Future studies are currently underway to focus on outcome endpoints measured by the incidence of cardiotoxicity, occurrence of cancer therapeutics held due to cardiovascular complications, and cardiovascular death. These studies are being conducted through the IRB-supported cardio-oncology registry to assess the impact of the novel care model on patient outcomes.

## Conclusions

**Fig. 8:**
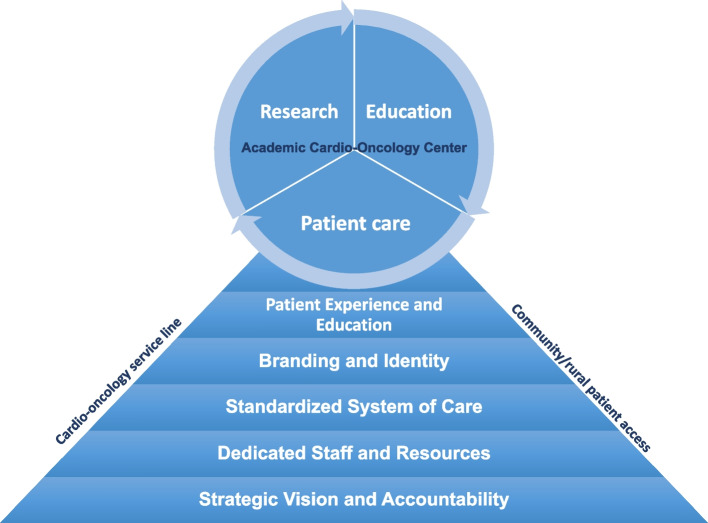
Novel integrated academic cardio-oncology center model. In the setting of the highly specialized area and relatively limited patient population in cardio-oncology, the establishment of the service line care model has significantly improved patient access and expanded the patient population, providing a strong foundation for research and education in this field. This has subsequently facilitated the advancement of cardio-oncology as a whole, enabling academic cardio-oncology centers to further their efforts in research and education

The cardio-oncology service line, which incorporates key infrastructure elements centered on a standardized system of care, is a feasible and effective care model for improving cardio-oncology care quality, patient access, and health equity in large, multi-hospital health systems. It can be used in conjunction with academic cardio-oncology programs to improve the overall efficacy of cardio-oncology healthcare for this patient population. Furthermore, due to the highly specialized nature of this area and the relatively limited patient population, the expanded patient population resulting from this care model can establish a strong foundation for patient care, supporting academic research and education (Fig. 8). This, in turn, can facilitate the advancement of the cardio-oncology field as a whole.

## Data Availability

Not applicable.
